# Physical Activity, Psychological and Functional Outcomes in Non-Ambulatory Stroke Patients during Rehabilitation—A Pilot Study

**DOI:** 10.3390/jcm11247260

**Published:** 2022-12-07

**Authors:** Marcin Błaszcz, Nina Prucnal, Krzysztof Wrześniewski, Szymon Pasiut, Piotr Mika, Małgorzata Kucia, Beata Stach, Marcin Woźniak, Elżbieta Mirek

**Affiliations:** 1 Institute of Clinical Rehabilitation, University of Physical Education in Krakow, 31-571 Krakow, Poland; 2 Non-Public Healthcare Facility “Pasternik”, 32-085 Modlniczka, Poland; 3 Emotion and Perception Lab, Institute of Psychology, Faculty of Philosophy, Jagiellonian University, Ingardena 6, 30-060 Krakow, Poland; 4 Department of Psychology, University of Physical Education in Krakow, 31-571 Krakow, Poland; 5 Musculoskeletal Rehabilitation Centre in “Krzeszowice”, 32-065 Krzeszowice, Poland; 6 Department of Physiotherapy, Faculty of Health Sciences, Jagiellonian University Collegium Medicum, 31-126 Krakow, Poland

**Keywords:** stroke, physical activity, self-efficacy, rehabilitation, non-ambulatory, functional outcomes, quality of life, depression, acceptance of illness

## Abstract

Despite the extensive literature on stroke rehabilitation, there are few studies that comprehensively show non-ambulatory stroke patients. The aim of the study was to explore the dynamics of the change in physical activity (PA), psychological and functional outcomes, and the correlation between them in non-ambulatory patients during early in-patient post-stroke rehabilitation. Measurements were taken on 21 participants at the beginning of and 6 weeks post-conventional rehabilitation with the Barthel Index (BI), Berg Balance Scale (BBS), Trunk Control Test (TCT), Stroke Impact Scale (SIS), General Self-Efficacy Scale, Stroke Self-Efficacy Questionnaire (SSEQ), the original scale of belief in own impact on recovery (BiOIoR), Hospital Anxiety and Depression Scale, Acceptance of Illness Scale and when the patient could walk—Time Up & Go and 6 Minute Walk Test. Daily PA was assessed over 6 weeks using a Caltrac accelerometer. Only outcomes for BI, BBS, TCT, SIS, and SSEQ significantly improved 6 weeks post-rehabilitation. PA energy expenditure per day significantly increased over time (*p* < 0.001; effect size = 0.494), but PA only increased significantly up to the third week. PA change was correlated with BiOIoR post-treatment. Self-efficacy in self-management mediated improvement in SIS. The BiOIoR and confidence in self-management could be important factors in the rehabilitation process.

## 1. Introduction

Stroke is the third reason for long-term disability, with 5 million new disabled people every year [1]. In 2019, there were over 12 million stroke incidents, 101 million prevalent strokes, and 143 million disability-adjusted life-years due to stroke [2]. Every third person who survives a stroke suffers from depression after its occurrence [3]. Despite the development of medicine, 8.6% of stroke survivors could not walk after 12 weeks of rehabilitation [4]. Physical activity (PA), i.a., by stimulating neuroplasticity improves patients functioning (especially during the window of enhanced neuroplasticity early after stroke, but also at a later time [5]) [6,7]. Despite these benefits, patients are still inactive and alone for the majority of the time during hospitalization, even in a comprehensive stroke unit [8]. In turn, time in bed in the early phase is associated with poor functional outcomes 3 and 6 months later [9,10]. PA in the stroke population is undoubtedly an important and frequently researched factor. There are many studies on PA in the stroke population [11]. However, most of the research [11] and guidelines do not focus on the non-ambulatory stroke population, as indicated in 2022 in a systematic review [12]. In turn, non-ambulatory stroke survivors are at the highest risk of inactivity, which is the greatest challenge to the healthcare system [9,13]. For example, in the latest Cochrane review of interventions for reducing sedentary behavior in people with stroke, most of them were able to walk and stand on their own [14]. None of the 10 trials focused only on non-ambulatory stroke patients. The authors indicated the need to include participants who are unable to stand or ambulate independently in further research [14]. Moreover, none of the studies included in Physical Activity after Stroke: A Systematic Review and Meta-Analysis recruited exclusively non-ambulatory patients [15].

Besides PA, several other factors can influence the recovery process after a stroke. One of them is self-efficacy, which, according to Bandura [16], is the belief in one’s ability to perform certain tasks or behave in some way in order to achieve the intended goals. In turn, general self-efficacy is a general belief that everything that happens can be dealt with [17]. The level of self-efficacy may influence the results of recovery [18], the occurrence of depression [19,20,21], improvement of professional performance [22], quality of life, PA, daily activities [23] and mobility [20] after a stroke.

Another important factor in stroke rehabilitation is the locus of control (LOC). LOC may influence the recovery process [24], moderate the relationship between the impact of stroke and depressive symptoms [25], as well as mediate between social support and self-management [26]. According to the social learning theory [27], LOC can be attributed to internal factors or external factors. It is assumed that people with an internal LOC (who believe their outcomes are contingent upon their behavior) are more likely to take active responsibility for their health and strive harder to recover from health threats [24,27]. Although recovery LOC has been studied before [28], the importance of believing in its own impact on recovery has probably not been separately studied so far.

Taking into account the importance of not only PA among stroke patients but also the psychological factors that may influence the recovery process, an interdisciplinary study was conducted. The primary aim of the study was to explore the dynamics of change in physical activity, as well as psychological and functional outcomes, in non-ambulatory patients during early in-patient post-stroke rehabilitation. To the best of our knowledge, there is a lack of multifactorial studies connecting psychological and functional outcomes with continuous quantitative PA measurement focused on non-ambulatory stroke patients during in-patient rehabilitation. Therefore, the secondary aim was to test the hypotheses that belief in own impact on recovery, stroke-specific and generalized self-efficacy (as independent variables) are significantly associated with changes in physical activity, health-related quality of life (HRQL), acceptance of illness, anxiety, depression, and functional independence, mobility, balance, trunk control and walking ability (as dependent variables).

## 2. Materials and Methods

### 2.1. Study Design, Setting and Participants

It was a prospective observational pilot study involving consecutive stroke patients who were qualified for early post-stroke stationary rehabilitation in a rehabilitation hospital (ORNR Krzeszowice or NZOZ “Pasternik” in Modlniczka, Poland) between April 2019 and July 2021. The study was conducted with the consent of the Bioethics Committees at the Regional Chamber of Physicians in Krakow with the number: 127/KBL/OIL/2018 and according to the rules of the Helsinki Declaration. The trial was prospectively registered in the Australian New Zealand Clinical Trials Registry with the number: ACTRN12619000226101. The inclusion criteria were as follows: admitted to the rehabilitation unit within 3 months of stroke onset; age between 50 and 85 years; non-ambulatory patients defined as unable to walk without personal assistance [29] (the Functional Ambulation Category score ≤2 [30]) on admission to the project but being ambulatory before the stroke and Mini-Mental State Examination (MMSE) score ≥21, which according to the MMSE manual means slight disturbances in cognitive functions. The exclusion criteria were: moderate cognitive and severe perceptual or communication impairments (incl. severe aphasia and severe hemispatial neglect); all orthopedic, rheumatological, and other conditions that could affect the study results. All participants provided informed consent.

### 2.2. Procedures and Outcome Measurements

All patients underwent similar conventional rehabilitation including individualized sessions of physiotherapy, speech, occupational and psychological therapy and other usual care provided in the rehabilitation hospitals. Rehabilitation was carried out 5 days a week (with additional shorter therapy on Saturday) for 6 weeks. Physiotherapy lasted around 1 h and, depending on the patient’s needs, included neurophysiological methods, mobility, functional, balance and arm training. The therapy was mainly concentrated on basic activities, gait re-education, normalization of tonus muscle, stimulation of perception, sensation, alignment and motor control. Gait re-education was started when the patient achieved some postural control during standing. All functional and psychological measurements were carried out twice, within one week of admission (1) and after 6 weeks of rehabilitation (2).

1. Physical activity

The amount and dynamics of change in daily physical activity (PA) were measured as physical activity energy expenditure (PAEE). Daily PAEE was assessed using the Caltrac device, (Muscle Dynamics, Inc., Tarrance, CA, USA). The Caltrac is a hip-mounted, portable uniaxial accelerometer that reports energy expenditure based on the measurement of body acceleration [31,32,33]. Patients were asked to wear the device in an elastic band over the hip on the paresis side from getting up in the morning (around 8.30) to going to bed (for dependent patients around 17.30 due to daily routine related dressing up) daily throughout the 6 weeks rehabilitation. The measurement started within one week of admission. PAEE was recorded in kcal/d on each working day. In the study, only calories used during PA (PAEE) were reported. Patients took the device off when necessary, e.g., during bathing. When the patient did not wear the device for a significant part of the day or accidentally switched on the pedal mode or weightlifting mode on the Caltrac, such a measurement was not taken for analysis. No study was found in which non-ambulatory stroke patients wore a device measuring PA every day for such a long period of time. Due to the lack of knowledge of the acceptability of such a measurement, as well as the lack of other studies with exactly the same methodology as ours, this project was conducted as a pilot study.

2. Functional outcomes

Functional measurements were provided twice, on admission (1) and 6 weeks post-rehabilitation (2). Functional independence was measured with the Barthel Index (BI) which focuses on the basic activities of daily life. The scores from 0 to 20 indicate total dependence, 21–60 severe dependence, 61–90 moderate dependence, 91–99 slight dependence, and 100 total independence [34]. Balance was assessed by the Berg Balance Scale (BBS). The BBS assessed the performance of 14 functional tasks requiring maintaining balance on a scale of 0 to 4. The sum of 56 points indicates excellent balance [35]. The Trunk Control Test (TCT) was used to assess four simple aspects of trunk movement with scores from 0 to 100. The Stroke Impact Scale (SIS) was used to evaluate health-related quality of life and disability. SIS is a self-reported questionnaire with a 59-item assessing the impact of stroke on strength, function, mobility, emotion, communication, cognition, and participation, using a five-point difficulty Likert scale [36]. If the patient could walk without personal assistance after 6 weeks post-rehabilitation walking ability was measured. The risk of falling and walking ability were assessed with Time Up & Go (TUG)—time that a person takes to rise from a chair, walk three meters, turn around, walk back to the chair and sit down [37]. Walking endurance was measured with a 6 min Walk Test (6MWT) measuring distance walked over 6 min, with excellent test-retest reliability for the early stroke phase [38].

3. Psychological outcomes

Psychological measurements were also provided twice, on admission—baseline (1) and 6 weeks post-rehabilitation (2) for patients’ belief in their impact on recovery, stroke-specific self-efficacy, general self-efficacy, depression, anxiety, acceptance of illness and health-related quality of life. Because no tool was found that would measure only “belief in own impact on recovery” (BiOIoR), an original scale was constructed by psychologists and physiotherapists (presented in Table 1). The patients were asked to rank 5 different factors on which their recovery depends, from the most significant (5 points) to the least significant (1 point). Each factor could obtain one unique number of points from 1–5. There was 1 item indicating that recovery depends on the patient himself (related to an internal locus of control) and 3 items indicated that recovery depends on external factors (related to an external locus of control (Table 1). This original scale is partly related to the Recovery Locus of Control Scale [39]. This original scale is available as Supplementary Material (Document S1). Stroke-specific self-efficacy was measured with the Stroke Self-Efficacy Questionnaire (SSEQ) contains 13 items measuring the strength of confidence of stroke survivors in relation to activities (functional performance) and self-management. SSEQ is based on Bandura’s self-efficacy theory. It demonstrates good internal consistency (Cronbach Alpha 0.90) and criterion validity [40]. The General Self-Efficacy Scale (GSES) was used to assess generalized self-efficacy (confidence in one’s ability to succeed in specific situations). The GSES is a ten-item self-report scale, with higher scores (max 40) indicating a stronger belief that one’s own actions are responsible for successful outcomes [41]. The risk of depressive and anxiety disorders was measured with Hospital Anxiety and Depression Scale (HADS). HADS has two subscales (anxiety and depression) with the following norms: lack (0–7), possible cases (8–10), and definite cases (11–21) [42]. However, in the stroke population, there is a recommendation to lower the cut-off value to 7 points [43]. The Acceptance of Illness Scale (AIS) was used to measure the level of illness acceptance with a total score oscillating between 8 and 40. A higher score means better adjustment to illness, with fewer negative illness-related emotions [44].

### 2.3. Data Analysis

For the analysis of PA, firstly, the median PAEE was calculated for each individual patient for each week. Secondly, the mean of the medians was calculated for each week (from the 1st to the 6th week). Only measurements from working days were included in the analysis due to the frequent lack of data and not wearing Caltrac during the whole weekend. To assess the dynamics of PA changes, only the differences between consecutive weeks and between the 1st and 6th week were analyzed. Six-repeated-measures analyses of variance (ANOVA and MANOVA) were used to compare differences in PA over time. F tests were followed by Bonferroni tests. In order to determine the strength of the effect, the partial eta-squared (η^2^_P_) was calculated, which values of 0.01, 0.06, and 0.14 corresponding to the low, medium and large strength of the effect. The time of rehabilitation corresponded to the independent variable, and PAEE to the dependent variable.

All statistical analysis was performed with Statistica 13.1, except for PA and mediation analysis for which IBM SPSS Statistics ver. 26 was used. Descriptive measures are shown as mean, standard deviation (SD), 95% confidence interval, and frequency (percentage). The normality of data was verified using the Shapiro–Wilk test. For comparison of variables with normal distribution Student’s *t*-test was used. The Wilcoxon signed-rank test and Mann–Whitney U test were performed for comparison of variables diverged from a normal distribution, respectively. In correlation analysis, SSEQ, BiOIoR and GSES corresponded to independent variables, and PAEE, BI, TCT, 6MWT, TUG, SIS, AIS and HADS to dependent variables. To determine the relationship between measurable variables with normal distribution the Pearson’s correlation coefficient was used. Otherwise, the non-parametric Spearman rank order correlation coefficient was used. The size of correlation coefficients was interpreted as low 0.3–0.5, moderate >0.5–0.7, high >0.7–0.9 and very high >0.9–1 [45]. In the absence of a complete set of measurements (e.g., PA), available measurements were taken into account for analysis. In statistical analysis, missing data were removed in pairs. The mediation analyses were performed to verify the mediation effect of independent variables (baseline score of SSEQ in self-management, BiOIoR and GSES, and change in SSEQ in activity) on changes of dependent variables with the time of rehabilitation for PAEE, BI, SIS, and HADS depression subscale. Additionally, BI, TCT, BBS, depression, HRQL and age (as the potentially confounding variables) were also verified as mediators for PAEE. A bias-corrected confidence interval, based on 5000 bootstrapped samples and correction for heteroscedasticity, was provided for the tested mediators. In order to check the differences between nominal variables (e.g., sex, completion of study) cross tabs and chi-square were used. All calculations used standard statistical significance (*p* < 0.05).

## 3. Results

### 3.1. Participants’ Characteristics

**Figure 1:**
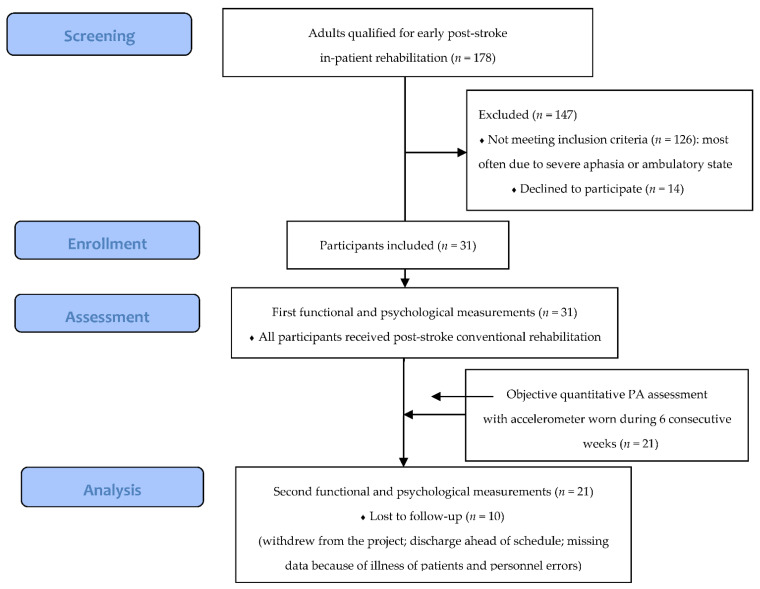
Flow diagram of study participation.

Of the 31 patients enrolled in the study, 21 patients were measured for PA over 6 weeks and completed 6-week rehabilitation combined with research observation. Ten participants dropped out of the study (Figure 1). Twenty-three patients completed the observation regarding walking abilities. Table 2 presents the demographic and clinical characteristics of the patients. In the group of participants who completed the study, there were statistically significant (*p* < 0.05) earlier time from stroke onset, more men, younger age and higher MMSE scores compared to those who dropped out.

### 3.2. Physical Activity over 6 Weeks Rehabilitation

**Figure 2:**
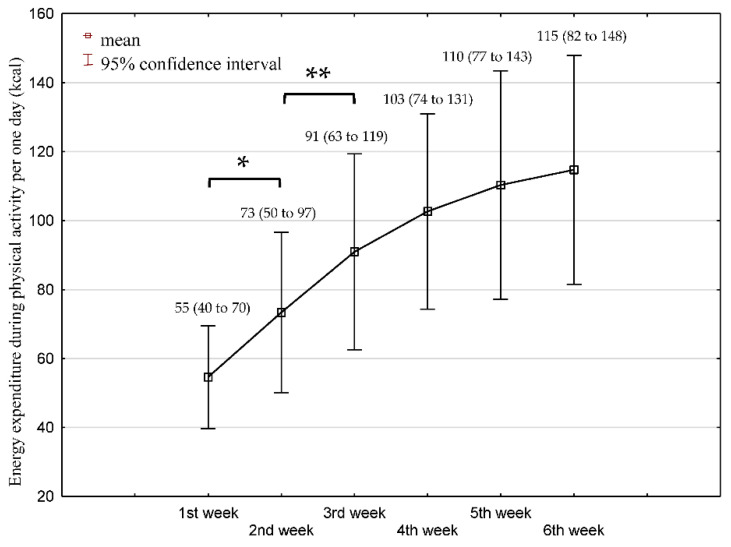
Mean and 95% confidence interval of physical activity energy expenditure (PAEE) (kcal/day) at each week over 6-week rehabilitation. *N* = 21. The mean PAEE was calculated from the median of individual patient outcomes during each week. A statistically significant difference between the following weeks is marked with bars and * *p* ≤ 0.05 or ** *p* ≤ 0.01.

The mean PAEE per day significantly increased over 6 weeks (*p* < 0.001; effect size as partial eta-squared = 0.494) from 54.4 to 114.7 kcal/day (110.1% increase). The mean difference between the first and sixth week was 60.1 kcal (95% CI 39.1 to 81.1). The differences were statistically significant between the first and second week (18.7 kcal; *p* = 0.019) and between the second and third week (17.6 kcal; *p* = 0.009). The differences between the third and fourth week (11.7 kcal); fourth and fifth (7.7 kcal); and fifth and sixth (4.4 kcal) were not significant (*p* > 0.05). There was a significant interaction between time of rehabilitation × sex (F = 5.71) = 3.975; *p* = 0.007; η^2^_P_ = 0.173. The women (*n* = 9) had lower daily PAEE compared to men (*n* = 12) in all timepoints: for the first week (25.4 ± 7.7 kcal vs. 76.6 ± 26 kcal), for the sixth week (57.7 ± 21 kcal vs. 157.5 ± 68.4 kcal) and for the difference between the first and sixth weeks (32.3 ± 17.4 kcal vs. 80.9 ± 50.5 kcal). The men had also a higher score in BI score (48.3 ± 18.5 vs. 29.4 ± 10.1; *p* = 0.01) and BBS score (15 ± 11.5 vs. 6.1 ± 2.2; *p* = 0.03) than women at baseline. Figure 2 presents the dynamics of PA change over 6 weeks of rehabilitation.

### 3.3. Functional and Psychological Outcomes at Baseline and 6 Weeks Post Rehabilitation

Table 3 presents functional and psychological outcomes at admission to the study and after 6 weeks of rehabilitation combined with research observation.

After 6 weeks of rehabilitation, 18 out of 23 patients were able to walk without physical assistance. The mean result for these patients for 6 Minute Walk Test was 166.31 ± 90.65 m (95% CI 121.23 to 211.39) and for Time Up & Go was 19.31 ± 10.0 s (95% CI 14.29 to 24.33).

### 3.4. Associations between Self-Efficacy, Belief in Own Impact on Recovery, and Functional and Psychological Outcomes and PA

Table 4 presents correlations between self-efficacy and belief in own impact on recovery, and functional and psychological outcomes and PA. For comparison of the studied variables with correlations related to health-related quality of life, the SIS score was also included. There were no significant correlations between independent variables (SSEQ, BiOIoR, GSES) as well as SIS, and dependent variables as daily PAEE during the first and sixth week, Δ Trunk Control Test, and HADS Depression subscale at baseline.

**Figure 3:**
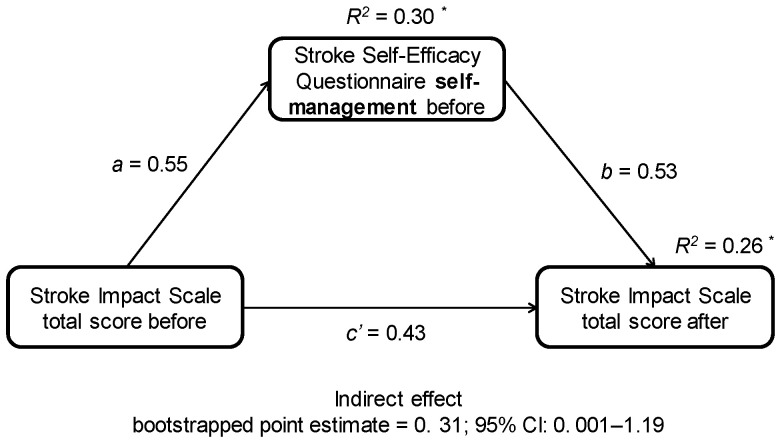
SIS total score at baseline (X) was positively related to SSEQ self-management at baseline (M) (a = 0.55; *p* < 0.05), and SSEQ self-management at baseline (M) was positively related to SIS total score post-6 weeks of rehabilitation (Y) while controlling SIS total score at baseline (X) (b = 0.53; *p* < 0.05). Thus, SIS total score baseline (X) indirectly (a × b) influenced SIS total score after (Y), through effect of SSEQ self-management at baseline (M) (bootstrapped point estimate = 0.31; 95% CI: 0.001–1.191). The direct effect of SIS total score baseline (X) on SIS total score after (Y) was not significant (c’ = 0.43; *p* > 0.05), which indicates that only indirect effect was significant in the model. *—*p* ≤ 0.05.

Regarding mediation analyses, only one mediation effect was significant. Self-efficacy in self-management at baseline significantly mediated the Stroke Impact Scale total score (Figure 3). There was no other significant mediation effect of independent variables as a mediator (SSEQ in self-management at baseline, BiOIoR at baseline, GSES at baseline, and change in SSEQ in activity) on changes of dependent variables with the time of rehabilitation for PAEE, BI, SIS total score, and HADS depression subscale. There was also no significant mediation effect when baseline scores for BI, TCT, BBS, depression, HRQL and age were verified as a mediator for changes in PAEE between the first and sixth week.

## 4. Discussion

### 4.1. Key Findings

The novelty of this study is its interdisciplinary focus on non-ambulatory patients during early in-patient rehabilitation connecting, psychological and functional outcomes with continuous quantitative physical activity measurement. Our data revealed that the participants’ PA significantly increased over 6 weeks of rehabilitation. PA increased from week to week, but significant changes were observed only up to the third week. The difference between PA in the first and sixth week correlated only with belief in own impact on recovery after 6 weeks of rehabilitation. Patients achieved significant improvement in all functional tests, and in stroke-specific self-efficacy and health-related quality of life. The BiOIoR was related with better health-related quality of life, acceptance of illness, stroke-specific and general self-efficacy, and lower risk of depression.

### 4.2. Physical Activity

The mean PAEE in our participants (54.4 in the first to 114.7 kcal/day in the sixth week) was low compared to other studies, which, however, mostly include ambulatory stroke patients [15,46,47]. Nevertheless, in Rand et al., study individuals with chronic stroke living in the community showed similar PAEE with a median of 98.1 kcal/day despite the fact that they were less disabled than our participants [48]. Recent studies also confirm that patients spend most of their time in bed. The Kunkel et al. results indicated poor PA during hospitalization. Stroke patients spent 94% of their time sitting/lying, 4% in standing and 2% walking [49]. In another study with 65 severe stroke patients, the mean proportion of time in upright activity was 0.9%, 25.7% in sitting out of bed, and 72% time in bed [50]. In our study, PA significantly increased over time. In turn, in the Kramer et al. study, activity levels also were low in an acute stroke ward but did not significantly change between baseline and four weeks post-stroke [51]. Likewise, according to Fini et al.’s review, including 5306 subjects, stroke survivors spent long periods inactive and sedentary throughout all stages following stroke, particularly during the acute phase. Although PA increased within the first 3 months post-stroke, then plateaued. The authors indicated that the behavior patterns established within the first 3 months post-stroke may dictate long-term PA habits [47]. In our study, analogously, the dynamics of PA growth flattened in the middle of observation (after 3 weeks). Despite the probable motor recovery, the increase in PA had slowed down after the third week. Due to that, the patients could lose many potential benefits of additional PA. We found that it is a big challenge for post-stroke rehabilitation to constantly activate the patients to use their potential of the newly acquired motor abilities. The recent meta-analyses showed that regular PA in stroke patients could prevent or reduce depressive symptoms [52]; improve cardiorespiratory fitness, muscle strength, walking capacity [53], bone mineral density [54] and quality of life [55]; reduce the risk of recurrent stroke [56]; and stimulate neuroplasticity [6]. In opposition to our results, in Rand et al.’s study, the PA was associated with better HRQL [48]. Due to the positive effect of PA on functional outcomes, these data are also relevant from a walking ability perspective. Lord et al. observed that more than one-third of stroke patients could be unable to walk unsupervised after being discharged home [57]. A meta-analysis including 25 thousand stroke patients indicated the significant effect of intensive high repetitive task-oriented, and task-specific training in all phases of poststroke physiotherapy [58]. These results show how important it is to accelerate PA post-stroke by therapists, especially in non-ambulatory patients.

In our study, men had significantly higher PAEE than women in all timepoint measurements and higher change in PAEE between the first and sixth week. However, it could be because men also had significantly higher functional independence and balance at baseline than women.

### 4.3. Functional and Psychological Outcomes during Rehabilitation

Patients achieved significant improvement in all functional measurements (BI, TCT, BBS and physical domain of SIS). The mean difference in functional independence (37.8 points) was higher than minimal clinically important differences (MCID) for inpatient rehabilitation for BI (35 points) [59]. The participants also achieved higher mean improvement in balance (23.6 points) than MCID for BBS (13.5 points) [60]. The patients also statistically significantly improved their self-efficacy in activity and self-management, although in our study only usual care was used, which is less effective in improving stroke-specific self-efficacy than self-management programs [61]. Moreover, we observed a significant improvement in six domains of SIS (strength, memory and thinking, emotions, ADL, mobility and hand function) and the perception of recovery. These results are similar to the previous study, in which there also were no significant changes over time in the SIS communication domain [62]. In turn, a slight deterioration in participation was due to in-patient rehabilitation limited work, travels, family duties, etc. Despite the significant improvements after 6 weeks of rehabilitation in most SIS domains, the impact of stroke on patients should be monitored longitudinally because it could get worst with time [63].

In opposition to the functional outcomes, SIS and SSEQ, the changes in other variables were not significant. We could observe a statistically insignificant decrease in the level of depression, anxiety, belief in external impact on recovery, and general self-efficacy as well as an increase in the level of the acceptance of illness and belief in own impact on recovery over time of the rehabilitation.

The results regarding depression, despite the insignificance of improvement, are similar to the previous study which showed a decrease in depression intensity [64]. We believe the insignificance of our results was because of the small sample size, among which were patients with low depression symptoms outcomes. Moreover, our study did not target any interventions in relation to the reduction in depressive and anxiety symptoms, which as indicated in the previous studies, are efficient in their role [65].

We observed a decrease in outcomes for external impact on recovery and an increase in internal impact on recovery. These changes, although statistically insignificant (maybe due to the small sample size and ceiling effect), represent a positive trend.

We have to mention that the decrease in our results of general self-efficacy is not in line with the previous research. One of the possible explanations for that is the lack of social support, to which our patients had limited access during their rehabilitation. The role of social support was indicated as a factor that may influence the changes in the level of self-efficacy after stroke [66].

### 4.4. Associations between Independent and Dependent Variables

Regarding the relationships between the studied variables, a high correlation coefficient (*r* > 0.7) was found between BiOIoR during the first measurement (1) and SSEQ in the self-management subscale during the second measurement (*r* = 0.75; *p* < 0.001). We also found this level of correlation coefficient (*r* > 0.7) between the SIS total score (1) and the SSEQ total score during the second measurement (*r* = 0.72; *p* < 0.001). Moreover, the results of BiOIoR during the second measurement (2) were moderately correlated (*r* > 0.55) with SIS total score (2) and with SSEQ (especially: total score of SSEQ (2), SSEQ in activity (2) and in self-management (1)). As we have shown, the belief in own impact on recovery could be positively related to an increase in PA and psychological aspects of functioning.

The BiOIoR (belief in own impact on recovery) is included in the Recovery Locus of Control Scale [39] and Multidimensional Health Locus of Control (MHCL) where it is treated as a part of the locus of control term. According to the authors’ knowledge, BiOIoR has not previously been investigated separately. Because previous studies were related in general to the health locus of control, not just BiOIoR, there might be differences between their results and ours. For example, the low level of locus of control may play a role in depression occurrence after a stroke, whereas the types of these beliefs (either external or internal) might make a difference [67]. Moreover, the moderating role of health-related locus of control on the relationship between stroke-related quality of life and post-stroke depressive symptoms occurrence has been found [25]. Additionally, in contrast to our results, Mohd et al. reported that internal recovery locus of control after stroke might be a predictive factor of 18% of the physical functioning [28]. Likewise, in previous research, the internal locus of control was a propitious factor for improving post-stoke recovery outcomes [67] and everyday activities [68]. While considering the aspects of health locus of control, it is worth noticing that the correlation between it and the acceptance of illness among stroke patients has not been shown before. In turn, our study showed the correlation between BiOIoR and acceptance of illness. We think that it seems to be worth measuring and studying the possibility of increasing the BiOIoR level during the rehabilitation process as it might be related to both PA and psychological outcomes, including acceptance of the illness and possibly depression.

Our results suggest that self-efficacy in self-management could have a positive indirect mediating effect on improving the health-related quality of life. In our study, stroke-specific self-efficacy in different aspects (either SSEQ total score, activity or self-management subscale) was also correlated with HRQL, functional independence, depression or acceptance of illness. These correlations are compatible with the systematic review of Jones and Riazi, in which high self-efficacy after stroke has a positive effect on the quality of life, perception of health, depression, everyday activities, and to some extent, physical functioning [23]. In turn, a meta-analysis including over 400 stroke patients showed that self-management programs improve quality of life and self-efficacy [61].

Additionally, speaking about general self-efficacy, its low level might be a warning sign for clinicians. General self-efficacy was found to correlate, but not mediate with the post-stroke quality of life level [69] and might influence a change in the intensity of depressive symptoms [21] as well as physical functioning and fulfilling everyday activities [21,23]. Furthermore, Rogowska et al. reported that general self-efficacy fully mediated the relationship between the health locus of control and life satisfaction [70].

Acceptance of the disease at baseline correlated with the initial stroke-specific self-efficacy, and baseline stroke disability and quality of life. It could indicate that a higher level of illness acceptance may be related to better subjective quality of life and a belief in the ability to manage the rehabilitation process. Moreover, the baseline level of disease acceptance correlated with the post-rehabilitation measurement of self-efficacy in self-management, which may mean that disease acceptance may play a role in the patient’s ability to cope with new life tasks during stroke recovery. These results are consistent with other studies indicating the existence of a relationship between self-efficacy and disease acceptance [66,71] as well as the impact of acceptance on the rehabilitation process [72]. Moreover, the continuous effects of improvement of disease acceptance and life satisfaction over time after stroke have been found [73].

Moreover, our results indicated that the higher levels of depression post-6-weeks, but not at the beginning of the rehabilitation, correlated negatively with self-efficacy in self-management, belief in own impact on recovery, general self-efficacy and HRQL. The change in depression symptoms severity after 6 weeks was negatively correlated with self-efficacy in self-management and general self-efficacy at baseline. These correlations are in line with previous research in which depression negatively correlated with general self-efficacy [19,74], self-efficacy related to stroke [20], locus of control [25] and functional status [74,75]. Therefore, special attention should be paid to increasing self-efficacy due to its association with reducing the severity of post-stroke depression.

For inference, it is also important that despite many significant correlations, none but one of the mediations studied was sufficient strength to conclude the influence of the examined factors on other dependent variables. Despite the significant correlation between BiOIoR post-6 weeks and change in PAEE, there was no mediation effect of BiOIoR neither at baseline nor post-6 weeks on changes in PAEE. There was also no other significant mediation effect of SSEQ in self-management, BiOIoR and GSES at baseline, and change in SSEQ in activity on changes of dependent variables (PAEE, BI, depression or SIS except for self-efficacy self-management). There was also no significant mediation effect of potentially confounding factors such as BI, TCT, BBS, depression, HRQL and age on changes in PAEE.

Our study revealed that it could be important to examine belief in own impact on recovery and ways that can increase it, reinforcing that belief among stroke patients. As we have shown, it correlates with better PA improvement, stroke-specific self-efficacy, acceptance of illness, and decreasing the negative impact of stroke on patients’ lives. In addition, it might be important to monitor the progress of physical activity and enhance patients to use their gained functional abilities and increase their PA. Moreover, our study may indicate the value of increasing self-efficacy in self-management to prevent or minimize the negative consequences of stroke. Therefore, strategies for increasing self-efficacy among stroke patients should continue to be developed.

### 4.5. Limitations

Our study has some limitations. Some participants dropped out due too to the number of questionnaires and PA continuous measurement, which had further consequences. Firstly, due to the small sample size, the statistical analysis of mediations was limited and could underestimate the strength and significance of mediations. Secondly, this small sample size did not allow for the reliable performance of multiple linear regression analysis. These limitations mean that only the correlations between SSEQ, BiOIoR, and GSES with functional and psychological outcomes, as well as PA were shown in this study, but not the cause-and-effect relationships. Taking into account described difficulties, it is worth including the mentioned dropout effect while planning the next experiments. Another limitation is the use of the original, but not standardized, scale for measuring belief in own impact on the recovery. However, firstly, to the authors’ knowledge, there is no scale that examines this belief separately. Secondly, we wanted to construct a short tool that could later be used in practice. Nevertheless, in future research, this scale might be either developed psychometrically or replaced with a different tool. The next limitation of the study was the possible disruptive impact of the COVID-19 pandemic on patient outcomes. Unfortunately, the size of the group before the pandemic was too small to reliably compare these groups in terms of the studied outcomes. What is more, the PAEE for any given task is higher in stroke compared with the general non-disabled population. The accelerometers underestimate PAEE in stroke patients, probably slightly less for sedentary/light activities [76] that are more typical for non-ambulatory stroke survivors. Nevertheless, the aim of our study was primarily to analyze the dynamics of changes in physical activity during 6 weeks of rehabilitation, and not to estimate the size of PAEE itself. Owing to all of these reasons, the received outcomes should be taken with caution. However, it is a pilot study, so this research topic should be studied with a larger sample size.

## 5. Conclusions

Physical activity increased from week to week, but significant changes were observed only up to the third week. This indicates the need for constant activation of patients to use their potential. Patients could achieve significant improvement in functional independence, trunk control, balance and in stroke-specific self-efficacy and health-related quality of life after 6 weeks of conventional rehabilitation. The psychological outcomes such as general self-efficacy, depression, anxiety and acceptance of illness could not improve significantly during this time with usual care. In the rehabilitation process, it is worth considering the belief in own impact on recovery and self-efficacy in self-management, as they could be associated with better psychophysical functioning of stroke patients.

## Figures and Tables

**Table 1:** Belief in Own Impact on Recovery scale.

INSTRUCTION: Rate what your recovery depends on, on a scale of 1 to 5. Where 1 is the least important factor and 5 is the most important factor. You can only assign each grade once.	
from me (my commitment, motivation, strategies for coping with stroke) ^iloc^	Rate: …
from a doctor, physical therapist, psychologist and other forms of therapy ^eloc^	Rate: …
from a family, relatives, my surroundings ^eloc^	Rate: …
from a disease progression ^eloc^	Rate: …
other (please specify if there are any other factors not listed on the scale)	Rate: …

^iloc^—factor related to an internal locus of control; ^eloc^—factor related to an external locus of control.

**Table 2:** Baseline characteristics of study subjects (*N* = 31).

Age (Years)	Gender (Female/Male)	Time since Stroke (Days)	Hemiplegic Side (R/L)	Ischemic/ Hemorrhagic Stroke	Mini-Mental State Examination	Before/during the COVID-19 Pandemic
72.3	18/13	33.3	13/18	29/2	25.6	11/20
±6.5	58.1%	±25.9	41.9%	93.5%	±3.1	35.5%

Data are presented as the mean ± standard deviation or proportion.

**Table 3:** Study outcomes at the beginning, 6 weeks post-rehabilitation, and within-group differences.

Outcome	Baseline (Mean ± SD)	Post 6 Weeks (Mean ± SD)	Change (Mean ± SD)	*p*-Value	Change CI (95%)
Barthel Index	41.7 (17.3)	79.6 (17.9)	**37.8 (19.6)**	**<0.001**	**(29.3; 46.3)**
Trunk Control Test	66.6 (26.9)	88.7 (16.2)	**22.2 (21.7)**	**<0.001**	**(12.8; 31.5)**
Berg Balance Scale	10.8 (9.4)	34.3 (12.3)	**23.6 (11)**	**<0.001**	**(18.2; 27.7)**
HADS					
anxiety	7.6 (3.2)	6.6 (3.5)	−1 (3.2)	0.165	(−2.4; 0.4)
depression	6.1 (4.2)	5.3 (4.3)	−0.8 (2.7)	0.234	(−2.1; 0.4)
total score	13.8 (6.2)	12 (6.6)	−1.8 (4.6)	0.098	(−3.9; 0.3)
SSEQ					
activities	4 (3.8)	13.5 (5.7)	**9.5 (6)**	**<0.001**	**(6.8; 12.2)**
self-management	8.8 (4.4)	10.5 (3.9)	**1.7 (3.3)**	**0.036**	**(0.1; 3.2)**
total score	12.9 (6.1)	23.8 (9)	**10.9 (6.4)**	**<0.001**	**(7.3; 14.5)**
Acceptance of Illness Scale	23.3 (7.9)	27 (7.9)	3.7 (1.6)	0.131	(−1.2; 8.5)
Belief in own impact on recovery (from 1 to 5)	4 (1.3)	4.5 (1.1)	0.5 (0.9)	0.059	(0; 0.9)
Belief in external impact on recovery (from 1 to 5)	2.7 (0.3)	2.7 (0.3)	−0.1 (0.3)	0.272	(−02; 0.1)
GSES	33.1 (6)	30.9 (7.6)	−2.2 (6.4)	0.139	(−5.1; 0.7)
Stroke Impact Scale					
total score	341.4 (61.1)	504.6 (118.2)	**163.1 (99.5)**	**<0.001**	**(117.8; 208.4)**
Strength	25.8 (20.8)	57.1 (22.5)	**31.3 (19.9)**	**<0.001**	**(22.6; 39.9)**
Memory and thinking	73.2 (18.3)	86.5 (13.8)	**13.3 (16)**	**0.002**	**(6.2; 20.5)**
Emotions	67.3 (18.3)	75.1 (16.5)	**7.8 (12.4)**	**0.01**	**(2.3; 13.3)**
Communication	80.5 (22.3)	89.5 (15.1)	8.9 (21.8)	0.068	(−0.7; 18.6)
ADL	22.4 (13.4)	54.6 (21.2)	**32.2 (24.7)**	**<0.001**	**(21.5; 42.8)**
Mobility	14.1 (8.4)	54.1 (24.6)	**40.1 (24.5)**	**<0.001**	**(29.5; 50.7)**
Hand function	8.7 (12.9)	32.4 (33.9)	**23.7 (27.1)**	**<0.001**	**(12; 35.4)**
Participation	45.9 (26.9)	44.5 (27.3)	−1.4 (20.2)	0.745	(−10.4; 7.5)
Perception of recovery (%)	23.9 (16.7)	57.6 (14.9)	**33.7 (21.1)**	**<0.001**	**(24.6; 42.8)**

Statistically significant differences are bolded. ADL—Activities and daily living; CI—confidence interval; GSES—General Self-Efficacy Scale; HADS—Hospital Anxiety and Depression Scale; SD—standard deviation; SSEQ—Stroke Self-Efficacy Questionnaire.

**Table 4:** Correlation coefficients between stroke-specific and general self-efficacy, belief in own impact on recovery, and functional and psychological outcomes, and PA.

Variables	Stroke Self-Efficacy Questionnaire						Belief in Own Impact on Recovery		General Self- Efficacy Scale			Stroke Impact Scale Total Score		
	Total Score		Activity		Self-Management									
	1	2	1	2	1	2	1	2	1	2	Δ	1	2	Δ
Belief in own impact on recovery (1)	0.32	0.64 **	−0.02	0.56 *	0.55 **	0.75 ***	-	0.56 *	0.37 *	0.32	−0.06	0.17	0.58 **	0.41
Belief in own impact on recovery (2)	0.12	0.23	−0.30	0.13	0.49 *	0.40	0.56 *	-	0.36	−0.03	−0.510 *	0.06	0.20	0.05
Stroke Impact Scale total score (1)	0.37 *	0.38	0.23	0.29	0.29	0.39	0.17	0.06	0.33	0.47 *	0.33	-	0.54 *	0.03
Stroke Impact Scale total score (2)	0.65 **	0.72 ***	0.19	0.69 ***	0.62 **	0.64 **	0.58 **	0.20	0.19	0.58 **	0.51 *	0.54 *	-	0.86 ***
Barthel Index (1)	0.20	0.01	0.24	0.02	0.03	0.02	0.07	0.38	0.09	−0.13	−0.10	0.45 *	0.04	−0.18
Barthel Index (2)	0.43 *	0.56 *	0.19	0.61 **	0.40	0.52 *	0.34	0.20	0.19	0.13	0.13	0.33	0.77 ***	0.58 **
Barthel Index Δ	0.18	0.46	−0.12	0.48 *	0.20	0.27	0.05	−0.02	0.13	0.16	0.17	0.03	0.51 *	0.68 ***
Berg Balance Scale Δ	0.24	0.47	−0.02	0.35	0.24	0.32	−0.07	−0.01	0.29	0.23	0.20	0.13	0.53 *	0.54 *
Time Up & Go	0.13	−0.48	0.12	−0.55 *	0.06	−0.37	0.08	0.08	−0.01	−0.43	−0.67 **	−0.15	−0.59 *	−0.53 *
6-Minute Walk Test	0.18	0.54	0.11	0.61 **	0.13	0.43	0.26	0.06	−0.07	0.33	0.58 *	0.42	0.69 **	0.60 *
HADS Depression subscale (2)	−0.12	−0.34	0.44 *	−0.31	−0.52 *	−0.45 *	−0.44 *	−0.48 *	−0.41	−0.53 *	−0.09	−0.32	−0.44 *	−0.36
HADS Depression subscale Δ	−0.40	−0.36	0.00	−0.37	−0.56 **	−0.35	−0.43	−0.32	−0.44 *	−0.22	−0.04	−0.16	−0.43	−0.41
Acceptance of Illness Scale (1)	0.48 **	0.36	0.37	0.26	0.32	0.56 **	0.14	0.05	0.24	0.40	0.12	0.38 *	0.34	0.05
Acceptance of Illness Scale (2)	−0.10	0.22	−0.37 *	0.24	0.17	0.32	0.33	0.45 *	0.16	0.06	−0.15	−0.24	−0.10	0.02
mean daily physical activity Δ 6th-1st week	0.02	0.36	−0.24	0.32	0.27	0.31	0.15	0.49 *	0.08	0.15	0.08	0.10	0.33	0.23

Statistically significant correlations are bolded. * *p* ≤ 0.05; ** *p* ≤ 0.01; *** *p* ≤ 0.001; Δ—the difference between baseline and post-6-weeks rehabilitation score; (1)—Baseline; (2)—post 6 weeks of rehabilitation; HADS—Hospital Anxiety and Depression Scale; SSEQ—Stroke Self-Efficacy Questionnaire. The correlations with Stroke Impact Scale are presented for a comparison with the correlations with independent variables.

## Data Availability

The data presented in this study are available upon request from the corresponding author.

## Supplementary Materials

The following supporting information can be downloaded at: https://www.mdpi.com/article/10.3390/jcm11247260/s1, Document S1: Belief in Own Impact on Recovery scale.

## References

[1] Feigin V.L., Krishnamurthi R.V., Parmar P., Norrving B., Mensah G.A., Bennett D.A., Barker-Collo S., Moran A.E., Sacco R.L., Truelsen T. (2015). Update on the Global Burden of Ischemic and Hemorrhagic Stroke in 1990-2013: The GBD 2013 Study. Neuroepidemiology.

[2] Feigin V.L., Stark B.A., Johnson C.O., Roth G.A., Bisignano C., Abady G.G., Abbasifard M., Abbasi-Kangevari M., Abd-Allah F., Abedi V. (2021). Global, Regional, and National Burden of Stroke and Its Risk Factors, 1990-2019: A Systematic Analysis for the Global Burden of Disease Study 2019. Lancet Neurol..

[3] Hackett M.L., Yapa C., Parag V., Anderson C.S. (2005). Frequency of Depression after Stroke. Stroke.

[4] Chu C.-L., Lee T.-H., Chen Y.-P., Ro L.-S., Hsu J.-L., Chu Y.-C., Chen C.-K., Pei Y.-C. (2022). Recovery of Walking Ability in Stroke Patients through Postacute Care Rehabilitation. Biomed. J..

[5] Ballester B.R., Maier M., Duff A., Cameirão M., Bermúdez S., Duarte E., Cuxart A., Rodríguez S., Mozo R.M.S.S., Verschure P.F.M.J. (2019). A Critical Time Window for Recovery Extends beyond One-Year Post-Stroke. J. Neurophysiol..

[6] Mackay C.P., Kuys S.S., Brauer S.G. (2017). The Effect of Aerobic Exercise on Brain-Derived Neurotrophic Factor in People with Neurological Disorders: A Systematic Review and Meta-Analysis. Neural Plast..

[7] Carey L., Walsh A., Adikari A., Goodin P., Alahakoon D., De Silva D., Ong K.L., Nilsson M., Boyd L. (2019). Finding the Intersection of Neuroplasticity, Stroke Recovery, and Learning: Scope and Contributions to Stroke Rehabilitation. Neural Plast..

[8] Kārkliņa A., Chen E., Bērziņa G., Stibrant Sunnerhagen K. (2021). Patients’ Physical Activity in Stroke Units in Latvia and Sweden. Brain Behav..

[9] Askim T., Bernhardt J., Salvesen Ø., Indredavik B. (2014). Physical Activity Early after Stroke and Its Association to Functional Outcome 3 Months Later. J. Stroke Cerebrovasc. Dis..

[10] Askim T., Bernhardt J., Churilov L., Fredriksen K.R., Indredavik B. (2013). Changes in Physicalactivity and Related Functional and Disability Levels in the First Six Months after Stroke: A Longitudinal Follow-up Study. J. Rehabil. Med..

[11] Thilarajah S., Mentiplay B.F., Bower K.J., Tan D., Pua Y.H., Williams G., Koh G., Clark R.A. (2018). Factors Associated With Post-Stroke Physical Activity: A Systematic Review and Meta-Analysis. Arch. Phys. Med. Rehabil..

[12] Church G., Ali A., Smith C.L., Broom D., Sage K. (2022). Examining Clinical Practice Guidelines for Exercise and Physical Activity as Part of Rehabilitation for People with Stroke: A Systematic Review. Int. J. Environ. Res. Public Health.

[13] Kubo H., Kanai M., Nozoe M., Inamoto A., Taguchi A., Mase K., Shimada S. (2022). Different Association between Physical Activity and Physical Function According to Walking Independence in Hospital-Based Rehabilitation Program Patients with Sub-Acute Stroke. Clin. Neurol. Neurosurg..

[14] Saunders D.H., Mead G.E., Fitzsimons C., Kelly P., van Wijck F., Verschuren O., Backx K., English C. (2021). Interventions for Reducing Sedentary Behaviour in People with Stroke. Cochrane Database Syst. Rev..

[15] Field M.J., Gebruers N., Shanmuga Sundaram T., Nicholson S., Mead G. (2013). Physical Activity after Stroke: A Systematic Review and Meta-Analysis. ISRN Stroke.

[16] Bandura A. (1993). Perceived Self-Efficacy in Cognitive Development and Functioning.Pdf. Educ. Psychol..

[17] Lewin A., Jöbges M., Werheid K. (2013). The Influence of Self-Efficacy, Pre-Stroke Depression and Perceived Social Support on Self-Reported Depressive Symptoms during Stroke Rehabilitation. Neuropsychol. Rehabil..

[18] Marks R., Allegrante J.P., Lorig K. (2005). A Review and Synthesis of Research Evidence for Self-Efficacy-Enhancing Interventions for Reducing Chronic Disability: Implications for Health Education Practice (Part II). Health Promot. Pract..

[19] Volz M., Voelkle M.C., Werheid K. (2019). General Self-Efficacy as a Driving Factor of Post-Stroke Depression: A Longitudinal Study. Neuropsychol. Rehabil..

[20] Korpershoek C., van der Bijl J., Hafsteinsdottir T.B. (2011). Self-Efficacy and Its Influence on Recovery of Patients with Stroke: A Systematic Review. J. Adv. Nurs..

[21] Torrisi M., De Cola M.C., Buda A., Carioti L., Scaltrito M.V., Bramanti P., Manuli A., De Luca R., Calabrò R.S. (2018). Self-Efficacy, Poststroke Depression, and Rehabilitation Outcomes: Is There a Correlation?. J. Stroke Cerebrovasc. Dis..

[22] Nott M., Wiseman L., Seymour T., Pike S., Cuming T., Wall G. (2021). Stroke Self-Management and the Role of Self-Efficacy. Disabil. Rehabil..

[23] Jones F., Riazi A. (2010). Self-Efficacy and Self-Management after Stroke: A Systematic Review. Disabil. Rehabil..

[24] Bonetti D., Johnston M. (2008). Perceived Control Predicting the Recovery of Individual-Specific Walking Behaviours Following Stroke: Testing Psychological Models and Constructs. Br. J. Health Psychol..

[25] Zirk M., Storm V. (2019). Subjective Stroke Impact and Depressive Symptoms: Indications for a Moderating Role of Health-Related Locus of Control. Front. Psychiatry.

[26] Lei T.T., Han H.M., Liu X.J. (2020). Multiple Mediation Effects of Health Locus of Control and Hope on the Relationship between Stroke Patients’ Social Support and Self-Management. Front. Nurs..

[27] Rosenstock I.M., Strecher V.J., Becker M.H. (1988). Social Learning Theory and the Health Belief Model. Health Educ. Behav..

[28] Mohd Zulkifly M.F., Ghazali S.E., Che Din N., Desa A., Raymond A.A. (2015). The Ability of Recovery Locus of Control Scale (RLOC) and Post-Traumatic Stress Symptoms (PTSS) to Predict the Physical Functioning of Stroke Patients. Malaysian J. Med. Sci..

[29] Lloyd M., Skelton D.A., Mead G.E., Williams B., van Wijck F. (2018). Physical Fitness Interventions for Nonambulatory Stroke Survivors: A Mixed-Methods Systematic Review and Meta-Analysis. Brain Behav..

[30] Holden M.K., Gill K.M., Magliozzi M.R., Nathan J., Piehl-Baker L. (1984). Clinical Gait Assessment in the Neurologically Impaired Reliability and Meaningfulness. Phys. Ther..

[31] Balogun J.A., Martin D.A., Clendenin M.A. (1989). Calorimetric Validation of the Caltrac® Accelerometer during Level Walking. Phys. Ther..

[32] Gebruers N., Vanroy C., Truijen S., Engelborghs S., De Deyn P.P. (2010). Monitoring of Physical Activity after Stroke: A Systematic Review of Accelerometry-Based Measures. Arch. Phys. Med. Rehabil..

[33] Plasqui G., Westerterp K.R. (2007). Accelerometers: An Evaluation against Doubly Labeled Water. Obesity.

[34] Mahoney F.I., Barthel D.W. (1965). Functional Evaluation: The Barthel Index. Md. State Med. J..

[35] Blum L., Korner-Bitensky N. (2008). Usefulness of the Berg Balance Scale in Stroke Rehabilitation: A Systematic Review. Phys. Ther..

[36] Vellone E., Savini S., Fida R., Dickson V.V., Melkus G.D.E., Carod-Artal F.J., Rocco G., Alvaro R. (2015). Psychometric Evaluation of the Stroke Impact Scale 3.0. J. Cardiovasc. Nurs..

[37] Johansen K.L., Stistrup R.D., Schjøtt C.S., Madsen J., Vinther A. (2016). Absolute and Relative Reliability of the Timed “Up & Go” Test and “30second Chair-Stand” Test in Hospitalised Patients with Stroke. PLoS ONE.

[38] Fulk G.D., Echternach J.L., Nof L., O’Sullivan S. Clinometric Properties of the Six-Minute Walk Test in Individuals Undergoing Rehabilitation Poststroke. Physiother. Theory Pract..

[39] Partridge C., Johnston M. (1989). Perceived Control of Recovery from Physical Disability: Measurement and Prediction. Br. J. Clin. Psychol..

[40] Jones F., Partridge C., Reid F. The Stroke Self-Efficacy Questionnaire: Measuring Individual Confidence in Functional Performance after Stroke. J. Clin. Nurs..

[41] Schwarzer R., Jerusalem M. (1995). Generalized Self-Efficacy Scale. J. Weinman, S. Wright, M. Johnston, Meas. Health Psychol. A user’s portfolio. Causal Control. Beliefs.

[42] Zigmond A.S., Snaith R.P. (1983). The Hospital Anxiety and Depression Scale. Acta Psychiatr. Scand..

[43] Wichowicz H.M., Wieczorek D. (2011). Badanie Przesiewowe Depresji Poudarowej z Użyciem Hospital Anxiety and Depression Scale (HADS). Psychiatr. Pol..

[44] Felton B.J., Revenson T.A., Hinrichsen G.A. (2001). AIS-Acceptance of Illness Scale. Meas. tools Promot. Health Psychol..

[45] Mukaka M.M. (2012). Statistics Corner: A Guide to Appropriate Use of Correlation Coefficient in Medical Research. Malawi Med. J..

[46] Haeuber E., Shaughnessy M., Forrester L.W., Coleman K.L., Macko R.F. (2004). Accelerometer Monitoring of Home- and Community-Based Ambulatory Activity after Stroke. Arch. Phys. Med. Rehabil..

[47] Fini N.A., Holland A.E., Keating J., Simek J., Bernhardt J. (2017). How Physically Active Are People Review and Quantitative Synthesis. Phys. Ther..

[48] Rand D., Eng J.J., Tang P.F., Hung C., Jeng J.S. (2010). Daily Physical Activity and Its Contribution to the Health-Related Quality of Life of Ambulatory Individuals with Chronic Stroke. Health Qual. Life Outcomes.

[49] Kunkel D., Fitton C., Burnett M., Ashburn A. (2015). Physical Inactivity Post-Stroke: A 3-Year Longitudinal Study. Disabil. Rehabil..

[50] Hokstad A., Indredavik B., Bernhardt J., Ihle-Hansen H., Salvesen O., Seljeseth Y.M., Schüler S., Engstad T., Askim T. (2015). Hospital Differences in Motor Activity Early after Stroke: A Comparison of 11 Norwegian Stroke Units. J. Stroke Cerebrovasc. Dis..

[51] Kramer S.F., Churilov L., Kroeders R., Pang M.Y.C., Bernhardt J. (2013). Changes in Activity Levels in the First Month after Stroke. J. Phys. Ther. Sci..

[52] Eng J.J., Reime B. (2014). Exercise for Depressive Symptoms in Stroke Patients: A Systematic Review and Meta-Analysis. Clin. Rehabil..

[53] Lee J., Stone A.J. (2020). Combined Aerobic and Resistance Training for Cardiorespiratory Fitness, Muscle Strength, and Walking Capacity after Stroke: A Systematic Review and Meta-Analysis. J. Stroke Cerebrovasc. Dis..

[54] Borschmann K., Pang M.Y.C., Bernhardt J., Iuliano-Burns S. (2012). Stepping towards Prevention of Bone Loss after Stroke: A Systematic Review of the Skeletal Effects of Physical Activity after Stroke. Int. J. Stroke.

[55] Chen M., De Rimmer J.H. (2011). Effects of Exercise on Quality of Life in Stroke Survivors: A Meta-Analysis. Stroke.

[56] Brouwer R., Wondergem R., Otten C., Pisters M.F. (2021). Effect of Aerobic Training on Vascular and Metabolic Risk Factors for Recurrent Stroke: A Meta- Analysis. Disabil. Rehabil..

[57] Lord S.E., McPherson K., McNaughton H.K., Rochester L., Weatherall M. (2004). Community Ambulation after Stroke: How Important and Obtainable Is It and What Measures Appear Predictive?. Arch. Phys. Med. Rehabil..

[58] Veerbeek J.M., Van Wegen E., Van Peppen R., Van Der Wees P.J., Hendriks E., Rietberg M., Kwakkel G. (2014). What Is the Evidence for Physical Therapy Poststroke? A Systematic Review and Meta-Analysis. PLoS ONE.

[59] Castiglia S.F., Galeoto G., Lauta A., Palumbo A., Tirinelli F., Viselli F., Santilli V., Sacchetti M.L. (2017). The Culturally Adapted Italian Version of the Barthel Index (IcaBI): Assessment of Structural Validity, Inter-Rater Reliability and Responsiveness to Clinically Relevant Improvements in Patients Admitted to Inpatient Rehabilitation Centers. Funct. Neurol..

[60] Song M.-J., Lee J.-H., Shin W.-S. (2018). Minimal Clinically Important Difference of Berg Balance Scale Scores in People with Acute Stroke. Phys. Ther. Rehabil. Sci..

[61] Fryer C., Luker J., Mcdonnell M., Hillier S. (2016). Self Management Programmes for Quality of Life in People with Stroke. Cochrane Database Syst. Rev..

[62] Pucciarelli G., Brugnera A., Greco A., Petrizzo A., Simeone S., Vellone E., Alvaro R. (2021). Stroke Disease-Specific Quality of Life Trajectories after Rehabilitation Discharge and Their Sociodemographic and Clinical Associations: A Longitudinal, Multicentre Study. J. Adv. Nurs..

[63] Skoglund E., Westerlind E., Persson H.C., Sunnerhagen K.S. (2019). Self-Perceived Impact of Stroke: A Longitudinal Comparison between One and Five Years Post-Stroke. J. Rehabil. Med..

[64] Paolucci S., Iosa M., Coiro P., Venturiero V., Savo A., De Angelis D., Morone G. (2019). Post-Stroke Depression Increases Disability More than 15% in Ischemic Stroke Survivors: A Case-Control Study. Front. Neurol..

[65] Yu H.L., Cao D.X., Liu J. (2019). Effect of a Novel Designed Intensive Patient Care Program on Cognitive Impairment, Anxiety, Depression as Well as Relapse Free Survival in Acute Ischemic Stroke Patients: A Randomized Controlled Study. Neurol Res..

[66] Szczepańska-Gieracha J., Mazurek J. (2020). The Role of Self-Efficacy in the Recovery Process of Stroke Survivors. Psychol. Res. Behav. Manag..

[67] Gutierrez D., Wing J.J., Drum E., Boden-Albala B. (2022). Abstract WP81: The Association between Health-Related Locus Of Control And Post-Stroke Disability, Quality Of Life, And Depression. Stroke.

[68] Hamzah A. (2014). Sugiyanto Strengthening of Health Locus of Control Could Increase the Independence of Post Stroke Patients in Implementing the Daily Activities at Home. J. Nurs. Care.

[69] Minshall C., Ski C.F., Apputhurai P., Thompson D.R., Castle D.J., Jenkins Z., Knowles S.R. (2020). Exploring the Impact of Illness Perceptions, Self-Efficacy, Coping Strategies, and Psychological Distress on Quality of Life in a Post-Stroke Cohort. J. Clin. Psychol. Med. Settings 2020 281.

[70] Rogowska A.M., Zmaczyńska-Witek B., Mazurkiewicz M., Kardasz Z. (2020). The Mediating Effect of Self-Efficacy on the Relationship between Health Locus of Control and Life Satisfaction: A Moderator Role of Movement Disability. Disabil. Health J..

[71] Kobylańska M., Kowalska J., Neustein J., Mazurek J., Wójcik B., Bełza M., Cichosz M., Szczepańska-Gieracha J. (2019). The Role of Biopsychosocial Factors in the Rehabilitation Process of Individuals with a Stroke. Work.

[72] Guzek Z., Kowalska J. (2020). Analysis of the Degree of Acceptance of Illness among Patients after a Stroke: An Observational Study. Clin. Interv. Aging.

[73] van Mierlo M.L., van Heugten C.M., Post M.W.M., de Kort P.L.M., Visser-Meily J.M.A. (2015). Life Satisfaction Post Stroke: The Role of Illness Cognitions. J. Psychosom. Res..

[74] Amaricai E., Poenaru D. (2016). V The Post-Stroke Depression and Its Impact on Functioning in Young and Adult Stroke Patients of a Rehabilitation Unit. J. Ment. Health.

[75] Alghwiri A.A. (2016). The Correlation between Depression, Balance, and Physical Functioning Post Stroke. J. Stroke Cerebrovasc. Dis..

[76] Serra M.C., Balraj E., DiSanzo B.L., Ivey F.M., Hafer-Macko C.E., Treuth M.S., Ryan A.S. (2017). Validating Accelerometry as a Measure of Physical Activity and Energy Expenditure in Chronic Stroke. Top. Stroke Rehabil..

