# Supplementary material for: Physical Activity, Psychological and Functional Outcomes in Non-Ambulatory Stroke Patients during Rehabilitation—A Pilot Study
Source: J Clin Med. 2022 Dec 7;11(24):7260. doi: 10.3390/jcm11247260 (PMC9781234; doi:10.3390/jcm11247260)
Supplement: Supplementary file 1 [file jcm-11-07260-s001.zip › jcm-1975155-supplementary.pdf]

Document S1

Belief in Own Impact on Recovery Scale

MEASUREMENT …………………… (baseline/ post-treatment)

Patient's first and last name: ……………………………………………………………………………………….. Test date: ….……………………….
Name and surname of the therapist conducting the test: ………………………………………………………………………………………….
Comments: ……

Rate **what your recovery depends on**, on a scale of 1 to 5. Where **1** is **the least important** factor and **5** is **the most important** factor. You can only assign each grade once. You assign only one factor 5, only one factor 4, only one factor 3, etc.

- ☐ **from me** (my commitment, my motivation, my strategies for coping with the effects of a stroke)
- ☐ **from a doctor, physical therapist, psychologist and other form of therapy**
- ☐ **from a family, relatives, my surroundings**
- ☐ **from a disease progression**
- ☐ **other** (please specify if there are any other factors not listed on the scale)

  ………
